# Association between Triglycerides to High-Density Lipoprotein Cholesterol Ratio and Death Risk in Diabetic Patients with New-Onset Acute Coronary Syndrome: A Retrospective Cohort Study in the Han Chinese Population

**DOI:** 10.31083/j.rcm2306190

**Published:** 2022-05-26

**Authors:** Dongdong Shi, Le Wang, Hongliang Cong

**Affiliations:** ^1^Clinical College of Thoracic Medicine, Tianjin Medical University, 300070 Tianjin, China; ^2^Department of Cardiology, Tianjin Chest Hospital, 300222 Tianjin, China

**Keywords:** triglycerides to high-density lipoprotein cholesterol, type 2 diabetes mellitus, acute coronary syndrome, all-cause mortality, cardiac death, cardiovascular disease, retrospective

## Abstract

**Background and Aims:**

The incidence of diabetes mellitus has reached an alarming level. Cardiovascular disease (CVD) is the leading cause of mortality in diabetic patients. However, the association between ratio and survival outcomes in patients with diabetes mellitus (DM) and new-onset acute coronary syndrome (ACS) remains unknown. This study aimed to assess the association between the TG/HDLC ratio and the risk of death in diabetic patients with new-onset acute coronary syndrome in the Han Chinese population.

**Methods:**

Data in this study were retrospectively collected from January 2016 to December 2016 from patients with type 2 diabetes mellitus (T2DM) and new-onset ACS in Tianjin Chest Hospital. Patients were classified according to the baseline TG/HDLC ratio. Kaplan-Meier survival curves were used to demonstrate survival outcomes. Univariate and multivariate Cox proportional risk regression analyses were used to evaluate the hazard ratios and 95% confidence intervals (CIs) for the risk of death. Subgroup analysis was used to determine the presence of any interaction.

**Results:**

In total, 152 patients died, 98 of them from heart disease. The Kaplan-Meier survival curve showed that there were no significant differences for both all-cause and cardiac mortality between Median 1 and Median 2 in log-rank test. Multivariate Cox regression analyses revealed that the adjusted hazard ratio increased significantly (*p *< 0.05) with increasing median TG/HDLC for not only all-cause mortality and cardiac death, but also nonfatal stroke, fatal stroke and fatal MI. The association between the TG/HDLC ratio and the risks of all-cause mortality and cardiac death in diabetic patients with new-onset ACS was similar among subgroups (*p *> 0.05).

**Conclusions:**

An elevated TG/HDLC ratio (TG/HDLC >1.522) is associated with an increased risk of all-cause and cardiac death risks in diabetic patients with new-onset ACS. Therefore, TG/HDLC ratio may be a beneficial parameter to evaluate the prognosis of this high-risk population.

## 1. Introduction

Diabetes mellitus (DM) is a significant health problem. The prevalence of diabetes has constantly increased over the past few decades and has reached an alarming level [1]. The International Diabetes Federation (IDF) Diabetes Atlas 10th edition revealed more than 500 million people worldwide developed DM, and about one in ten adults was affected. Moreover, the number of diabetic patients has increased by 74 million in the last two years, highlighting the alarming increase in the global prevalence of diabetes [1]. The IDF speculated that this number would reach 783 million by 2045, and the proportion of adults with the disease could reach one in eight. Diabetes is also an important driver of global mortality [1]. The IDF also estimated that approximately 6.7 million adults would die from diabetes or its complications in 2021, accounting for more than one-tenth of the all-cause deaths worldwide and one in every five seconds due to diabetes [1].

Type 2 diabetes mellitus may affect more than 600 million people worldwide in the next 20 years [1]. It has a significant impact on survival and quality of life, especially in patients diagnosed at a younger age [1]. Although all complications of diabetes are significant, widespread cardiovascular disease remains the leading cause of morbidity and mortality in this population [2]. These amazing statistics highlight the urgent need for renewed attention to aggressive cardiovascular risk reduction in diabetic patients, especially those already suffering from acute coronary syndrome (ACS).

Although diabetic patients with ACS have high mortality, the relationship between the TG/HDLC ratio and the risk of death in patients with DM and new-onset ACS is unclear. Research on the TG/HDLC ratio has gradually increased, as this lipid parameter is closely related to many diseases. Several previous studies have indicated a positive correlation between the TG/HDLC ratio and hypertension [3, 4], insulin resistance [5, 6], metabolic syndrome [7, 8], and fatty liver [9, 10]. In addition, an elevated TG/HDLC ratio plays an important role in periodontal disease and renal insufficiency. Therefore, we carried out a retrospective cohort study to assess the association between the TG/HDLC ratio and the risk of death in diabetic patients with new-onset ACS.

## 2. Methods

### 2.1 Study Population

This is a retrospective cohort study involving patients admitted to Tianjin Chest Hospital between January 2016 and December 2016. A total of 1782 diabetic patients with new-onset ACS were enrolled in the study. ACS was subdivided into either non-ST-segment elevation myocardial infarction (MI), ST-segment elevation MI, or unstable angina pectoris. Twenty-two patients with incomplete follow-up data were excluded from the study. Based on a median TG/HDLC ratio, patients were divided into the following two groups: Median 1 (n = 880, TG/HDLC ≤1.522), Median 2 (n = 618, TG/HDLC >1.522). A total of 928 males and 832 females were enrolled in this analysis. The Institutional Review Board of Tianjin Chest Hospital approved this study. The study was a retrospective analysis of clinical data, so informed consent was not required.

### 2.2 Data Collection and Related Definitions

Clinical data, including sex, age, smoking status, history of hypertension, ACS types and duration of diabetes, were collected by trained technicians. Blood tests included total cholesterol (TC), high-density lipoprotein cholesterol (HDLC), low-density lipoprotein cholesterol (LDLC), triglycerides (TG), fasting plasma glucose (FPG), hemoglobinA1c (HbA1c), hypersensitive C-reactive protein (hs-CRP), troponin T, serum creatinine, and N-terminal pro-brain natriuretic peptide (NT-proBNP). All blood samples were collected intravenously and analyzed by the laboratory of Tianjin Chest Hospital using standard automated technologies. Cardiac ultrasound was used to measure left ventricular cardiac ejection fraction (LVEF); all the ultrasound reports were from Tianjin Chest Hospital. The glomerular filtration rate (eGFR) was derived using the MDRD equation. Body mass index (BMI) was calculated as ${\mathrm{weight/height}}^{2}$. The non-HDLC level was obtained by subtracting HDLC from TC. Major adverse cardiovascular events were defined as cardiac death, nonfatal MI, or nonfatal stroke. A patient’s survival status, alive or dead, was determined by telephone follow-up on a case-by-case basis. The cause of death was confirmed by phone.

### 2.3 Endpoint and Mortality Surveillance

The study’s endpoints included all-cause mortality and cardiac death. All-cause mortality was defined as death from any cause, including cardiac death and any other cause, such as cancerand stroke. Cardiac death was defined as MI, heart failure, and arrhythmia. Investigators were asked to follow up with patients at least once a year for the duration of the study which ended on February 23, 2021, except in the event of the patient’s death.

### 2.4 Statistical Analysis

The Kolmogorov–Smirnov test was used to determine whether the continuous variables conform to a normal distribution. If normally distributed, it was expressed as mean ± standard deviation and tested for significance using ANOVA. If skewed, the distribution was expressed in median and tested for significance using the Kruskal-Wallis test. The Kaplan-Meier survival curve demonstrated survival outcomes. Stepwise backwards Cox proportional hazards regression analysis was used to estimate hazard ratio (HR) and 95% CIs. The time-dependent Cox regression model was used to test whether the variables met the pH hypothesis, and then these variables were included in the multivariate Cox regression model. Univariable and multivariable analyses were performed using Cox regression analysis to evaluate the effects of TG/HDLC ratio on all-cause and cardiac mortality. Subgroup analysis of all-cause and cardiac mortality was performed according to age, sex, smoking status, hypertension, LDLC, and HbA1c. Differences between subgroup analyses were also compared using an interaction test. All two-sided *p*-values < 0.05 were considered statistically significant. All statistical analyses and charts were completed using the GraphPad Prism version 8.0.2 (GraphPad Prism, San Diego, CA, USA) and MedCalc version 20.0.4 (MedCalc Software Ltd, Ostend, Belgium).

## 3. Results

### 3.1 Baseline Characteristics

A total of 1760 diabetic patients with new-onset ACS were selected for analysis. Table 1 summarizes the baseline characteristics of patients in the two groups, which were based on a median split of the TG/HDLC ratio. Most variables were not statistically different between groups, including age, sex, smoking, hypertension, BMI, duration of Diabetes, LVEF, Lipoprotein(a) (Lp(a)), HbA1c, FPG, eGFR, troponin T, treatment strategies, aspirin, statin, β-blocker, ACEI/ARB, CCB, and nitrate, (*p *> 0.05). Significant variables between the two groups, included TG/HDLC ratio (*p *< 0.001), TC (*p *< 0.001), TG (*p *< 0.001), HDLC (*p *< 0.001), LDLC (*p *< 0.05), very low-density lipoprotein cholesterol (VLDL) (*p *< 0.001), N-terminal pro-brain natriuretic peptide (NT-proBNP) (*p *< 0.05), high-sensitivity C-reactive protein (hs-CRP) (*p *< 0.001), ACS types (*p *< 0.05) and Clopidogrel/Ticagrelor (*p *< 0.05). Moreover, TG and LDLC increased as the TG/HDLC ratio increased.

**Table 1.** **Baseline characteristics of included patients by median of TG/HDLC ratio**.

Variables		Total	Median 1	Median 2	*p*-value
No. at risk		1760	880	880	
Age, years		66.0 ± 6.7	66.1 ± 6.8	66.0 ± 6.7	0.774
Sex					0.390
	Female	832 (47.3%)	425 (48.3%)	407 (46.3%)	
	Male	928 (52.7%)	455 (51.7%)	473 (53.8%)	
Smoking					0.173
	ever or current	706 (40.1%)	339 (38.5%)	513 (58.3%)	
	never	1054 (59.9%)	541 (61.5%)	367 (41.7%)	
Hypertension		1354 (76.9%)	681 (77.4%)	673 (76.5%)	0.651
BMI, kg/$m^{2}$		25.6 ± 2.7	25.4 ± 2.9	25.6 ± 2.7	0.074
Duration of diabetes, months		8.0 (3.0–14.0)	8.0 (3.0–14.0)	8.0 (3.0–14.0)	0.540
LVEF, %		60 (56–64)	60 (56–63)	60 (56–64)	0.161
Laboratory findings					
TG/HDLC ratio		2.1 (1.8–2.9)	1.0 (0.8–1.3)	2.1 (1.8–2.9)	<0.001
TC, mmol/L		4.3 (3.5–5.0)	4.5 (3.8–5.3)	4.3 (3.5–5.0)	<0.001
TG, mmol/L		1.5 (1.1–2.1)	1.1 (0.9–1.4)	2.1 (1.7–2.7)	<0.001
HDLC, mmol/L		2.0 (0.9–4.8)	1.2 (1.0–1.3)	0.9 (0.8–1.1)	<0.001
LDLC, mmol/L		2.8 (2.1–3.5)	2.9 (2.3–3.6)	2.8 (2.1–3.5)	0.024
VLDL, mmol/L		0.5 (0.3–0.6)	0.4 (0.2–0.5)	0.5 (0.3–0.6)	<0.001
Lp(a), mmol/L		26.9 (10.7–75.3)	30.1 (12.9–75.5)	26.9 (10.7–75.3)	0.265
HbA1c, %		7.4 (6.1–9.3)	7.3 (6.6–8.3)	7.3 (6.6–8.3)	0.475
FPG, mmol/L		7.3 (6.1–9.3)	7.2 (5.9–9.1)	7.4 (6.1–9.3)	0.103
Hcy, µmol/L		12.7 (10.4–15.9)	12.5 (10.2–15.9)	12.8 (10.6–15.8)	0.142
eGFR, mL/min		92.5 ± 24.4	93.1 ± 24.0	92.5 ± 24.4	0.975
hs-CRP, mg/L		2.0 (0.9–4.8)	1.7 (0.7–4.9)	2.0 (0.9–4.8)	0.007
troponin T, μg/L		0.059 (0.025–0.095)	0.061 (0.026–0.098)	0.056 (0.023–0.092)	0.054
NT-proBNP, pg/mL		224.5 (99.9–631.6)	204.4 (89.1–612.0)	224.5 (99.9–631.6)	0.049
Treatment strategies					0.596
	Medication only	548 (31.3%)	284 (32.3%)	264 (30.0%)	
	PCI	1009 (57.2%)	496 (56.4%)	513 (58.3%)	
	CABG	203 (11.5%)	100 (11.4%)	102 (11.6%)	
ACS types					0.029
	Unstable angina	1379 (78.4%)	667 (75.8%)	712 (80.9%)	
	non-STEMI	162 (9.2%)	88 (10.0%)	74 (8.4%)	
	STEMI	219 (12.4%)	125 (14.2%)	94 (10.7%)	
Medications at discharge					
Aspirin		1697 (96.4%)	845 (96.0%)	852 (96.8%)	0.369
Statin		1667 (94.7%)	836 (95.0%)	831 (94.4%)	0.881
Clopidogrel/Ticagrelor		1386 (78.8%)	676 (76.8%)	710 (807%)	0.048
β-blocker		1129 (64.1%)	563 (64.0%)	566 (64.3%)	0.881
ACEI/ARB		1007 (57.2%)	506 (57.5%)	501 (56.9%)	0.810
CCB		491 (27.9%)	218 (24.8%)	254 (28.9%)	0.366
Nitrate		905 (54.1%)	450 (51.1%)	455 (51.7%)	0.812
Insulin		688 (39.1%)	342 (38.9%)	346 (39.3%)	0.845

Note: Continuous data are shown as mean standard deviation or median (interquartile range) and categorical data are shown as frequency (%). Abbreviations: BMI, body mass index; LVEF, left ventricle ejection fraction; TC, total cholesterol; TG, triglycerides; HDLC, high-density lipoprotein cholesterol; LDLC, low-density lipoprotein cholesterol; TG/HDLC ratio, triglycerides to high-density lipoprotein cholesterol ratio; VLDL, very low-density lipoprotein cholesterol; Lp(a), lipoprotein(a); HbA1c, hemoglobin A1c; Hcy, homocysteine; FPG, fasting plasma glucose; eGFR, estimated glomerular filtration rate; hs-CRP, high-sensitivity C-reactive protein; NT-proBNP, N-terminal pro brain natriuretic peptide; PCI, percutaneous coronary intervention; CABG, coronary artery bypass graft; ACEI, angiotensin-converting-enzyme inhibitor; ARB, angiotensin receptor blocker; CCB, calcium channel blocker.

### 3.2 Survival Curve

**Fig. 1.**
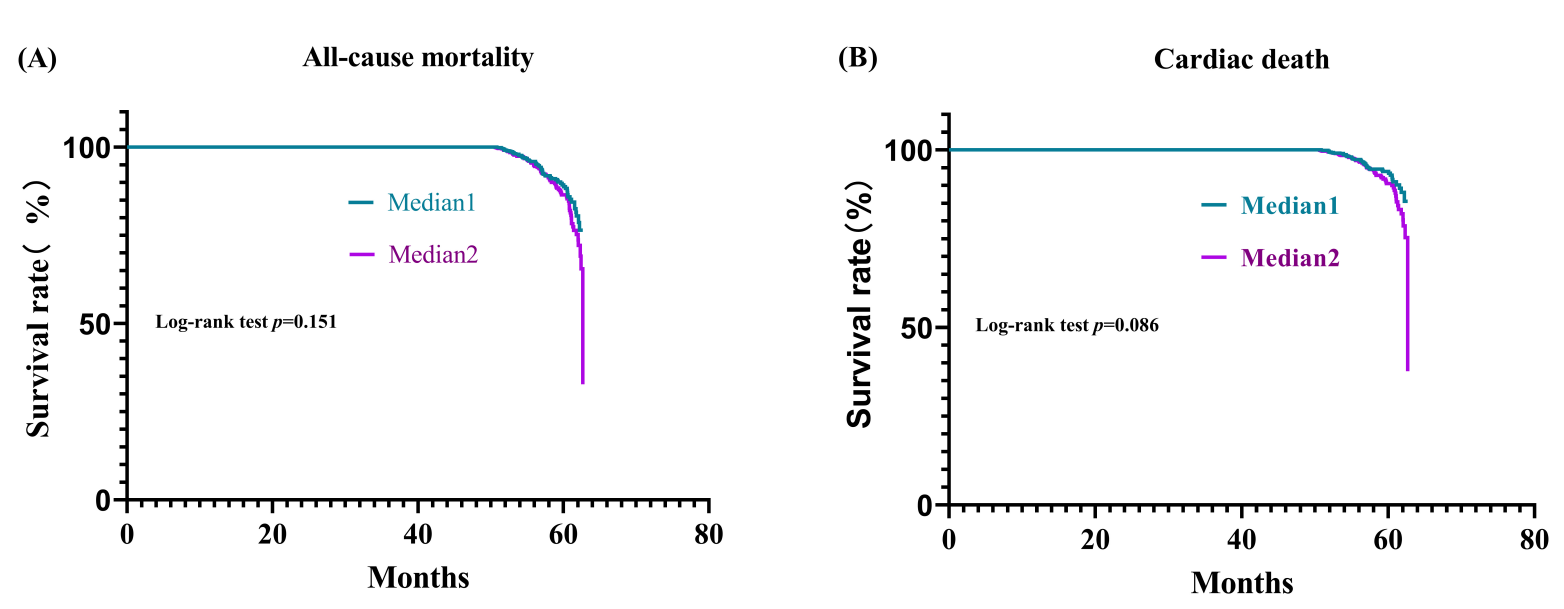
**Survival analyses**. (A) Kaplan-Meier survival curve for all-cause mortality across TG/HDLC ratio median. (B) Kaplan-Meier survival curve for cardiac mortality across TG/HDLC ratio median.

Fig. 1 shows the Kaplan-Meier survival curve for the risk of death. Time referred to the interval between admission and the last follow-up visit or patient death. All-cause mortality and cardiac death increased gradually after 50 months and increased almost vertically at approximately 62.5 months. But there were no significant differences for both all-cause and cardiac mortality between Median 1 and Median 2 in log-rank test.

### 3.3 Univariate and Multivariate Cox Regression Analysis

Table 2 shows the results of the Cox regression analysis. The TG/HDLC ratios were statistically significant after adjusting for confounders and for all-cause mortality, cardiac death, nonfatal stroke, fatal stroke, fatal MI and some non-cardiac death. However, the ratios were not statistically significant for nonfatal MI, sudden death and major adverse cardiovascular events after adjusting for confounders. Before adjustment, the risks of all-cause mortality and cardiac death between the two groups were similar. After adjusting for confounders, an increase in the TG/HDLC ratio was associated with an increased risk of cardiac death (*p *< 0.001) and all-cause death (*p* = 0.004).

**Table 2.** **Cox regression models evaluating the death risk and cardiovascular events according to TG/HDLC ratio**.

Endpoint		Events, n/total (%)	Crude HR (95% CI)	Crude *p*-value	Adjusted HR (95% CI)	Adjusted *p*-value
All-cause mortality		152/1760 (8.6%)		0.152		0.002
	Median 1	68/880 (7.7%)	1.00 (reference)		1.00 (reference)	
	Median 2	84/880 (9.5%)	1.26 (0.92–1.74)		1.90 (1.27–2.86)	
Cardiac death		98/1760 (5.6%)		0.088		<0.001
	Median 1	41/880 (4.7%)	1.00 (reference)		1.00 (reference)	
	Median 2	57/880 (6.5%)	1.42 (0.95–2.12)		2.44 (1.45–4.10)	
Nonfatal MI		77/1760 (4.4%)		0.714		0.513
	Median 1	40/880 (4.5%)	1.00 (reference)		1.00 (reference)	
	Median 2	37/880 (4.2%)	0.92 (0.58–1.46)		1.23 (0.67–2.26)	
Nonfatal stroke		435/1760 (24.7%)		0.498		0.004
	Median 1	227/880 (25.8%)	1.00 (reference)		1.00 (reference)	
	Median 2	208/880 (23.6%)	0.94 (0.78–1.13)		1.34 (1.10–1.64)	
Cardiac death plus nonfatal MI or nonfatal stroke		502/1760 (28.5%)		0.471		0.055
	Median 1	262/880 (29.8%)	1.00 (reference)		1.00 (reference)	
	Median 2	240/880 (27.3%)	0.94 (0.79–1.12)		1.20 (1.00–1.44)	
fatal MI		37/1760 (2.1%)		0.211		0.006
	Median 1	15/880 (1.7%)	1.00 (reference)		1.00 (reference)	
	Median 2	22/880 (2.5%)	0.92 (0.58–1.46)		3.22 (1.41–7.40)	
fatal stroke		15/1760 (8.5%)		0.013		0.013
	Median 1	2/880 (0.2%)	1.00 (reference)		1.00 (reference)	
	Median 2	13/880 (0.5%)	6.56 (1.48–29.05)		8.67 (1.58–47.50)	
sudden death		20/1760 (1.1%)		0.967		0.850
	Median 1	10/880 (1.1%)	1.00 (reference)		1.00 (reference)	
	Median 2	10/880 (1.1%)	1.02 (0.42–2.45)		1.13 (0.33–3.88)	
fatal stroke plus fatal MI or sudden death		72/1760 (4.1%)		0.028		0.004
	Median 1	27/880 (3.1%)	1.00 (reference)		1.00 (reference)	
	Median 2	45/880 (5.1%)	1.71 (1.06–2.76)		2.47 (1.34–4.54)	

Note: Abbreviations: HR, hazard ratio; CI, confidential interval; MI, myocardial infarction. Ajusted variables for all-cause mortality: smoking stauts, age, TG, NT-proBNP, eGFR, Hcy, FPG, LVEF, ACS types. Ajusted variables for cardiac death: smoking stauts, duration of diabetes, age, TG, NT-proBNP, eGFR, Hcy, FPG. Ajusted variables for fatal MI: smoking stauts, age, TG, NT-proBNP, LVEF, Hcy, FPG, troponin T, ACS types. Ajusted variables for fatal stroke: NT-proBNP, eGFR, troponin T, insulin, statin, Clopidogrel/Ticagrelor. Ajusted variables for fatal stroke plus fatal MI or sudden death: smoking stauts, age, TG, NT-proBNP, Hcy, troponin T, insulin, statin, ACS types.

### 3.4 Subgroup Analyses

**Fig. 2.**
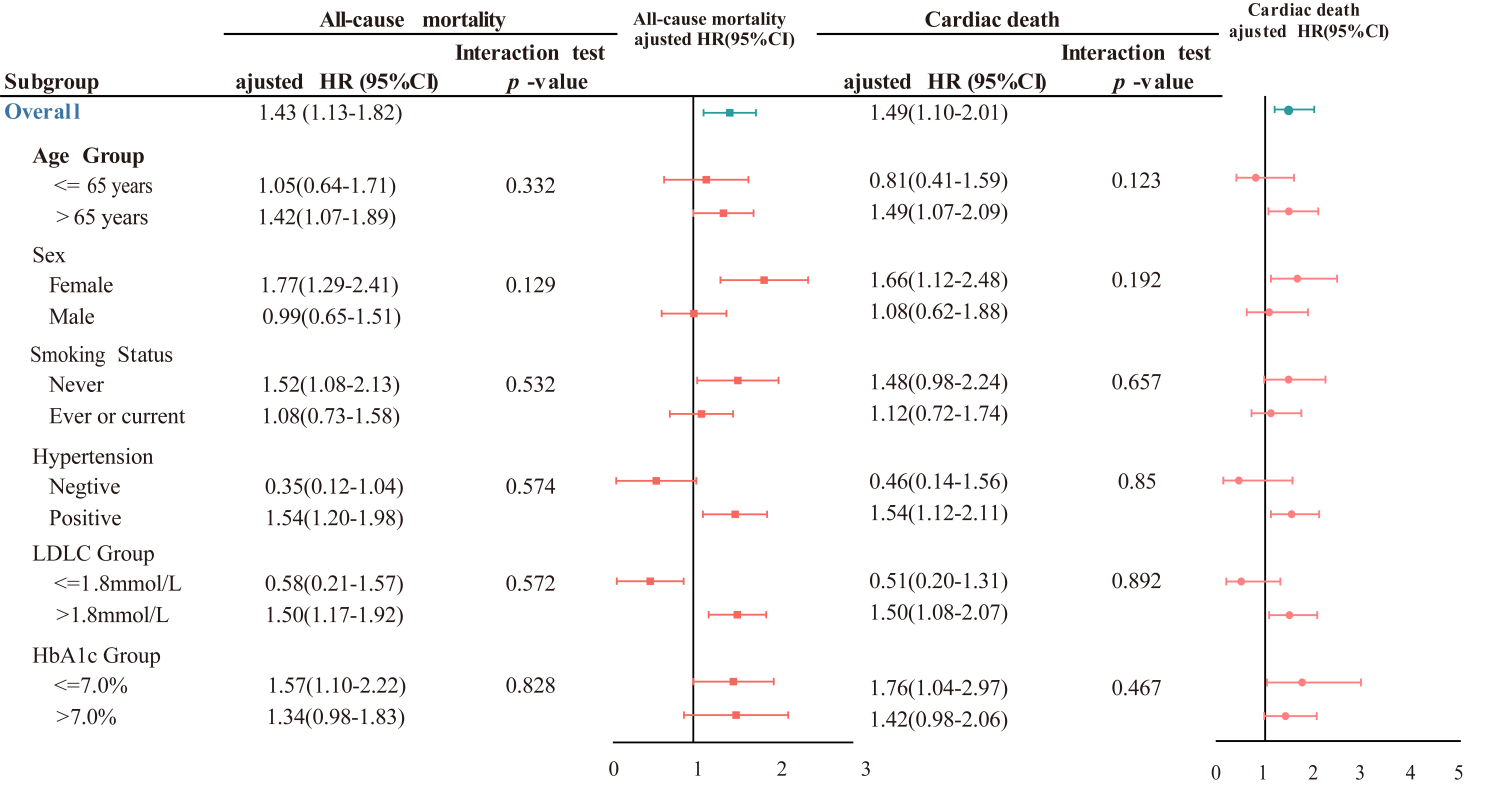
**Subgroup analyses**. Test the interactions in prespecified subgroups. The *p* values for interaction in all subgroups were more than 0.05. HR, hazard ratio; CI, confidential interval.

Fig. 2 illustrates the results of the subgroup analysis for all-cause and cardiac mortality. The TG/HDLC ratio was not statistically different in evaluating all-cause and cardiac death risks regarding age, sex, smoking status, hypertension, LDLC, and HbA1c (all of *p* values > 0.05 in subgroups).

## 4. Discussion

This study analyzed the association between the TG/HDLC ratio and the risks of all-cause mortality and cardiac death in diabetic patients with new-onset ACS. An elevated TG/HDLC ratio (TG/HDLC >1.522) is associated with an increased risk of all-cause and cardiac death risks in diabetic patients with new-onset ACS. Multivariate Cox regression analysis demonstrated the TG/HDLC ratio was a risk factor of all-cause and cardiac death. In the subgroup analysis, there was no statistical difference between the TG/HDLC ratio and all-cause and cardiac death risks in terms of age, sex, smoking status, hypertension, LDLC, and HbA1c.

There is an advantage to the TG/HDLC ratio to assess the risk of death in diabetic patients. High TG is a cardiovascular risk factor and has been associated with all-cause mortality and the incidence of coronary artery disease (CAD) events [11]. Several epidemiological studies have shown a significant relationship between serum HDLC concentration and CAD risk. The typical lipid profile of diabetes was high TG and low HDLC [11]. TG and HDLC were independent of each other, and in the absence of insulin resistance, a single lipid parameter did not reflect the actual status of plasma atherosclerosis and the risk of CAD. However, the TG/HDLC ratio combining both plasma atherosclerosis and CAD could better predict the risk of death in diabetic patients with new-onset ACS. It also appears to be a better indicator for primary and secondary prevention of cardiovascular diseases (CVDs) [12, 13, 14]. A previous study suggested that the TG/HDLC ratio had a better predictive value for mortality than individual lipid parameters [15]. In addition, a high TG/HDLC ratio was a good predictor of the extent of CAD [16, 17]. An elevated TG/HDLC ratio was an independent predictor of the long-term all-cause mortality in patients undergoing coronary angiography and was strongly associated with long-term risk of major adverse cardiovascular events [18]. Therefore, the TG/HDLC ratio assessment is of clinical value in diabetic patients with new-onset ACS.

The TG/HDLC ratio is associated with a residual risk of cardiovascular disease. In a certain proportion of patients taking oral statins, however, the risk of cardiovascular disease remains increased despite LDLC compliance. Both remnant lipoprotein particle cholesterol (RLPC) and LDLC are associated with the risk of ischemic heart disease (IHD) and MI [19]. A previous study showed that a residual cholesterol level ≥24 mg/dL was associated with an increased risk of atherosclerosis-associated disease regardless of the LDLC level [20]. Increased RLPC concentration was associated with an increased risk of all-cause mortality [21]. Using intravascular ultrasound, Bayturan *et al*. [22] found that LDL-C fell to an average of 58.4 mg/dL (1.5 mmol/L) in approximately twenty percent of intensively treated patients, but plaque numbers still increaed. RLPC explains part of the residual risk of all-cause mortality in patients with IHD [23]. However, no biological marker can quantify its level due to its apparent heterogeneity, lack of universally accepted definition, and absence of precise measurement methods. Although statins did not eliminate the residual risk of CVDs, Renato *et al*. [24] demonstrated that TG/HDLC was associated with residual cholesterol. Previous studies revealed that TG/HDLC ratio was closely associated with adverse cardiovascular events in patients with CAD [18, 25, 26]. A study found that the TG/HDLC ratio was a robust independent predictor of CAD, CVD, and all-cause mortality [27]. An elevated TG/HDLC ratio was reported to be a potentially useful predictor of future cardiovascular events in Chinese patients with DM and stable CAD [28]. Therefore, the TG/HDLC ratio assessment can be used clinically for risk stratification in patients receiving statin therapy.

The predictive value of the TG/HDLC ratio for cardiovascular events in diabetic patients is controversial. However, insulin resistance (IR) may be responsible for this controversy because it plays a critical role in cardiovascular events in diabetic patients. One study found that high TG and low HDLC levels were significant risk factors for coronary heart disease (CHD) only in the presence of IR [29]. Another study showed that the risk of major cardiovascular events was significantly greater in the presence of IR, regardless of whether triglyceride and HDL cholesterol levels were high or low [30]. Other studies have shown that IR at any level of obesity exacerbated the risk of developing CHD and T2DM [31]. The mechanisms by which insulin resistance promotes cardiovascular events in diabetic patients are as follows. (1) Triglyceride-enriched VLDL particles are hydrolyzed by lipoprotein lipase or hepatic lipase to produce small dense LDLC (sdLDLC) particles [32]; (2) In the presence of IR and high secretion of VLDL particles, these sdLDLC particles are usually present in high concentrations [33]; (3) Whereas sdLDLC particles are highly atherogenic, compared to normal LDL particles, they are more easily oxidized, have a higher affinity for the extracellular matrix, and have a higher degree of retention in the arterial wall [32]. In addition, the smaller the LDL, the less it binds to the LDL receptor, and the longer it resides in the circulation [32].

Summarizing the findings of the previous literatures, we found that the relationship between the TG/HDLC ratio and the risk of death in diabetic patients with new-onset ACS is unclear. Clarifying this relationship is extremely important to assess the prognosis of this high-risk population. This relationship has yet to be established in the published literature. Therefore, to clarify the relationship between the TG/HDL ratio and the risk of death in diabetic patients with new-onset ACS, we used Cox regression analysis and subgroup analysis to explore this relationship. We found that the TG/HDLC ratio was positively associated with the risk of death in diabetic patients with new-onset ACS.

There may be several potential mechanisms for the association between the TG/HDLC ratio and the risk of death in patients with DM and new-onset ACS: (1) Elevated TG level and reduced HDLC play a vital role in the progression of atherosclerosis, which may be related to the TG/HDLC ratio as a marker of LDL particle size [34]. Previous studies have reported that a high TG/HDLC ratio was strongly associated with elevated levels of small, dense LDLC, which is considered to be very atherogenic [35, 36, 37]. (2) The TG/HDLC ratio is significantly associated with insulin resistance in diabetic patients [38, 39, 40]. Furthermore, insulin resistance is associated with increased vulnerability of atherosclerotic plaque rupture resulting in ACS [41]. (3) The TG/HDLC ratio is related to the severity of atherosclerosis because the total plaque area is positively correlated with the TG/HDLC ratio [40]. (4) Hyperglycemia contributes to systemic macrovascular and microvascular disease in diabetic patients, including diabetic nephropathy, CAD, and ischemic stroke, which may be an additional risk for all-cause and cardiac death [42, 43, 44].

Several limitations of this study should be acknowledged: (1) Follow-up information was collected by telephone or electronic medical records. This information mainly included survival information. Baseline data after four years of follow-up were not collected. Because blood lipid levels varied by race, it was unclear whether these findings also apply to other races. (2) The complications and severity of new-onset ACS and DM differed, affecting the risks of all-cause mortality and cardiac death. (3) In this study, we found that overweight patients account for about half (54.83%), but obese patients account for smaller proportion (18.01%). The patients (LVEF >60%) account for about half (52.73%), but the patients (LVEF <50%) account for 12.67%.

Therefore, the overweight patients account for a large proportion, but the obese patients account for a small proportion. Besides, the patients (LVEF <50%) account for a small proportion. These patients may be a specific group, therefore the results of the study may be biased to some extent.

There are some reasons why non-obese patients develop diabetes as follows: (1) A genetic defect can lead to mitochondrial dysfunction. People with this genetic defect are unable to burn glucose or fatty acids efficiently, which can contribute to lipotoxicity and fat accumulation in muscle cells. (2) Non-alcoholic fatty liver is an independent predictor of type 2 diabetes, and a cause of insulin resistance and type 2 diabetes. (3) Chronic inflammation is an important mechanism that leads to insulin resistance in muscle, liver and fat cells.

As a new lipid-lowering drug, the proprotein convertase subtilisin/Kexin type 9 (PCSK9) inhibitor is gaining attention [45]. Therefore, we intend to study whether PCSK9 inhibitor can affect the TG/HDLC ratio to decreasethe risk of all-cause and cardiac death in diabetic patients with new-onset ACS.

## 5. Conclusions

An elevated TG/HDLC ratio (TG/HDLC >1.522) is associated with an increased risk of all-cause and cardiac death risks in diabetic patients with new-onset ACS. Therefore, TG/HDLC ratio may be beneficial to evaluate the prognosis of this high-risk population.

## Data Availability

The data related to the study findings can be requested from the corresponding author for appropriate reasons.

## Abbreviations

ACS, acute coronary syndrome; BMI, Body mass index; CAD, coronary artery disease; CVD, cardiovascular disease; CI, confidence interval; DM, diabetes mellitus; T2DM, type 2 diabetes mellitus; EGFR, estimated glomerular filtration rate; FPG, fasting plasma glucose; HbA1c, hemoglobinA1c; HR, hazard ratio; hs-CRP, hypersensitive C-reactive protein; IDF, International Diabetes Federation; IHD, ischemic heart disease; Lp(a), Lipoprotein(a); LVEF, left ventricular cardiac ejection fraction; MI, myocardial infarction; NT-proBNP, N-terminal pro brain natriuretic peptide; eGFR, PCSK9, proprotein convertase subtilisin/Kexin type 9; RLPC, remnant lipoprotein particle cholesterol; sdLDLC, small dense LDLC; TC, total cholesterol; TG, triglycerides; HDLC, high-density lipoprotein cholesterol; LDLC, low-density lipoprotein cholesterol; VLDL, very low-density lipoprotein cholesterol; Lp(a), lipoprotein(a); TG/HDLC, triglycerides to high-density lipoprotein cholesterol.
